# Safety study of sitagliptin combined with metoprolol on the treatment of elderly patients with type 2 diabetes mellitus and hypertension

**DOI:** 10.1515/med-2026-1538

**Published:** 2026-10-05

**Authors:** Xiaoqing Dai, Jiali Yu

**Affiliations:** General Practice, Tianshui-Wulin Sub-district Community Health Service Center, Hangzhou City, Zhejiang Province, China

**Keywords:** type 2 diabetes mellitus, hypertension, sitagliptin, metoprolol, safety

## Abstract

**Objectives:**

Sitagliptin is a standard therapeutic option for type 2 diabetes mellitus (T2DM), while metoprolol is an antihypertensive agent. This study aimed to assess the safety of their combination therapy in elderly patients with T2DM and hypertension.

**Methods:**

Medical data of 52 elderly patients with T2DM and hypertension from June 2021 to March 2024 were retrospectively collected. According to different treatment methods, 52 patients were categorized into the sitagliptin + metoprolol group (n=26) and the sitagliptin group (n=26). After a 12-week treatment period, the baseline characteristics, therapeutic efficacy and biochemical indicators were analyzed between groups.

**Results:**

After 12-week treatment, both groups exhibited significant improvements in therapeutic efficacy, immune-inflammatory status, coagulation function, and liver-kidney function (p<0.05). Compared with the sitagliptin group, the sitagliptin + metoprolol group showed better therapeutic efficacy, with lower FPG, HbA_1c_, SBP, and DBP levels (p<0.05). The combination group also achieved greater improvements in immune-inflammation, coagulation, and liver-kidney function (lower CRP, NLR, Fib, Scr and BUN, as well as higher PT and APTT) (p<0.05). There was no significant difference in incidence of adverse event between the two groups (p>0.05).

**Conclusions:**

Short-term observation indicates that sitagliptin combined with metoprolol can improve blood glucose, blood pressure, inflammation, coagulation, and liver-kidney function in elderly patients with T2DM and hypertension, without a significant increase in the risk of adverse reactions during short-term follow-up. However, as a single-center, small-sample retrospective study, the present findings should be interpreted as preliminary clinical evidence.

## Introduction

Type 2 diabetes mellitus (T2DM), the most common type of diabetes, refers to a chronic disease with insufficient insulin secretion and/or insulin resistance due to genetic and/or environmental factors, which leads to the increase of blood glucose level [1], 2]. T2DM usually occurs in middle-aged and elderly people over 40 years old, first-degree family members with a history of diabetes, as well as obese and overweight people [3]. T2DM is associated with multi-organ complications, affecting eyes, kidneys, nerves, heart and blood vessels [4]. Reportedly, hypertension frequently coexists with T2DM, which severely affects patients’ health outcomes [5]. Hence, there is a pressing need to develop effective treatment strategy to improve the prognosis of T2DM patients with hypertension.

At present, hypoglycemic drugs remain a mainstay of T2DM treatment, such as sitagliptin [6]. Sitagliptin, a potent and highly selective dipeptidyl peptidase-4 (DPP-4) enzyme inhibitor, can effectively improve blood glucose [7], suppress the DPP-4 activity to trigger insulin secretion, reduce pancreatic β cell apoptosis and decrease blood glucose level, thereby mitigating T2DM [7], 8]. Nevertheless, the therapeutic efficacy of sitagliptin in elderly patients with T2DM and hypertension has not been fully characterized.

Metoprolol, as a cardiac-selective β-blocker, is effective in managing hypertension and tachycardia, and improving vascular function [9], 10]. However, the combined effect of sitagliptin and metoprolol in elderly patients with T2DM and hypertension is still unknown.

To address this gap, we conducted a retrospective analysis of the medical records from elderly T2DM patients with hypertension who received either sitagliptin plus metoprolol or sitagliptin monotherapy, followed by a thorough analysis. The main objective is to evaluate the short-term efficacy and safety of the combination regimen in elderly T2DM patients with hypertension, thus providing preliminary observational data for clinical medication.

## Materials and methods

### Research design

In this retrospective study, the clinical data of elderly T2DM patients with hypertension were collected to evaluate the safety of sitagliptin combined with metoprolol. This study was approved by the Ethical Committee of Tianshui-Wulin Sub-district Community Health Service Center, Gongshu District, Hangzhou City (No. 2024-LL0815-01).

### Study objects

Medical data were retrospectively collected from elderly patients with T2DM and hypertension from June 2021 to March 2024 (n=52). All cases were divided into a combination group (sitagliptin + metoprolol, 26 cases) and a monotherapy group (sitagliptin alone, 26 cases) according to their prescribed clinical treatment plan, which were determined based on their blood pressure and blood glucose control levels, organ tolerance, and comorbidities. This study enrolled elderly patients with indications for the standard use of β-blockers, and selected metoprolol as a combined antihypertensive drug based on clinical diagnostic and treatment guidelines. A metoprolol monotherapy group and a placebo control group were not included for the following reasons: all subjects were diagnosed with T2DM and required glucose-lowering treatment. Both groups uniformly used sitagliptin for glycemic control, and there were no clinical cases solely receiving antihypertensive treatment without glucose-lowering therapy. The control group in this study consisted of patients with mild hypertension who only received sitagliptin for glycemic control and did not take antihypertensive drugs as prescribed, a grouping reflecting actual clinical diagnostic and therapeutic settings in the community. The biochemical test indicators of both groups before treatment and after 12 weeks of intervention were systematically organized and compared.

T2DM was diagnosed according to the “Guidelines for Diagnosis and Treatment of Diabetes in the Elderly in China (2024 Edition)”, meeting at least one of the following three criteria [11]: (1) fasting blood glucose ≥7.0 mmol/L; (2) 2-h postprandial blood glucose ≥11.1 mmol/L; (3) typical symptoms (drinking more, urinating more, eating more and losing weight) with random blood glucose ≥11.1 mmol/L.

Diagnostic criteria of hypertension refers to the “Guidelines for Prevention and Treatment of Hypertension in China (2024 Revision)”, with diagnostic thresholds defined as systolic blood pressure (SBP) ≥140 mmHg and/or diastolic blood pressure (DBP) ≥90 mmHg [12].

Inclusion criteria: (1) elderly patients diagnosed with T2DM complicated with hypertension; (2) normal consciousness level; (3) not taking other hypoglycemic drugs and antihypertensive drugs during the treatment period.

Exclusion criteria: (1) elderly patients allergic to sitagliptin or metoprolol; (2) incomplete clinical information; (3) severe dysfunction of liver, kidney and other organs before treatment; (4) clinical deterioration during the treatment period.

### Evaluation of the sample size

The sample size was assessed by the blood glucose indicator of fasting plasma glucose (FPG) [13]. The anticipated level of FPG was 9.29 ± 0.96 before treatment and 7.59 ± 0.66 after treatment. The sample size was calculated using PASS software (v21.0.3, NCSS Inc., USA) with parameters: power=90 %; α=0.05; dropout rate=25 %; and number of patients=1:1. Then, a minimum of 10 patients per group was required. Hence, 26 samples in each group were considered effective in the present study.

### Description of treatments

Elderly patients in the sitagliptin + metoprolol group received the combined treatments of sitagliptin and metoprolol for 12 weeks, while those in the sitagliptin group received monotherapy of sitagliptin for 12 weeks. The sitagliptin (oral tablet, 100 mg/day) and metoprolol (oral tablet, 47.5 mg/day) were obtained from Hangzhou Merck Sharp & Dohme (China) and AstraZeneca AB (Sweden), respectively.

### Collection and analysis of clinical data

#### Baseline characteristics

According to the medical records of elderly patients, the baseline characteristics were analyzed between the sitagliptin + metoprolol group and the sitagliptin group, including age, gender, body mass index (BMI), duration of diabetes, and duration of hypertension. This retrospective medical record analysis was limited by incomplete and non-uniform medical record system, including confounding indicators such as previous history of cardiovascular diseases, and concurrent use of other medications.

#### Therapeutic effect

The therapeutic efficacy on T2DM was assessed using FPG and glycosylated hemoglobin (HbA_1c_), while antihypertensive efficacy was evaluated by SBP and DBP. The decreases of FPG, HbA_1c_, SBP and DBP represented the improvements of T2DM and hypertension in elderly patients.

#### Biochemical indicators

The immune-inflammatory response-related indicators included C-reactive protein (CRP) and neutrophil to lymphocyte ratio (NLR). The coagulation function-related indicators comprised prothrombin time (PT), activated partial thromboplastin time (APTT) and fibrinogen (Fib). The liver function-related indicators incorporated alanine aminotransferase (ALT) and aspartate aminotransferase (AST). The kidney function was represented by serum creatinine (Scr) and blood urea nitrogen (BUN).

In brief, fasting venous blood (4 mL) from each elderly patient was collected, where NLR value, values of PT, APTT and Fib, and BUN level were assessed using the automatic blood cell analyzer (DP-H10, DyMind, China), full automatic blood coagulation instrument (STA Compact Max, Stago, France) and BUN assay kit (C013-2-1, Nanjing Jiancheng Bioengineering, China), respectively. After centrifugation, serum samples were collected, in which CRP, ALT, AST and Scr levels were severally evaluated using the CRP assay kit (H126-1-2), ALT assay kit (C009-2-1), AST assay kit (C010-2-1) and Scr assay kit (C011-2-1). These kits were all obtained from Nanjing Jiancheng Bioengineering (China). Finally, absorbance was read using a microplate reader (VLBL0TD2, Thermo Fisher Scientific, USA).

Fasting peripheral venous blood samples were collected from all patients in the morning before and after treatment for comprehensive serum biochemical tests. Blood pressure was measured uniformly after a 10-min rest in a supine position in the morning. Throughout this study, the same brand of electronic blood pressure monitor was used, and all blood samples were processed in the central laboratory department of our hospital using standardized testing reagents and an automatic biochemical analyzer.

This study presets the following indicators as the primary efficacy endpoints after 12 weeks of treatment: for glucose lowering, fasting plasma glucose (FPG) and glycosylated hemoglobin (HbA_1c_); for blood pressure lowering, systolic blood pressure (SBP) and diastolic blood pressure (DBP), while other indicators such as inflammation, coagulation, liver, and kidney function were secondary observation indicators.

### Statistical analysis

The data analysis was conducted using the SPSS 25.0 software (SPSS Inc., USA). Chi-square test was used for comparing categorical variables. For continuous variables that were normally distributed, data are expressed as mean ± standard deviation. The independent sample *t* test was used for between-group comparisons, and the paired sample *t* test was applied for within-group comparisons (pre- vs. post- treatment). Non-normally distributed continuous variables were presented by quartile method [M (P_25_-P_75_)], which were compared by Mann-Whitney *U* test between groups, and by the Wilcoxon signed rank test before and after treatment. Bilateral p<0.05 was deemed as significant difference.

## Results

### Baseline characteristics

The baseline characteristics (age, gender, BMI, duration of diabetes, and duration of hypertension) were listed in the Table 1. There were 26 elderly patients in the sitagliptin + metoprolol group (including 20 males and 6 females) and 26 elderly patients in the sitagliptin group (including 19 males and 7 females). The mean age was 65.19 ± 7.31 years in the sitagliptin + metoprolol group and 67.08 ± 7.67 years in the sitagliptin group. Moreover, the BMI was 25.83 ± 1.69 in the sitagliptin + metoprolol group and 25.07 ± 1.54 in the sitagliptin group. The average duration of diabetes in the sitagliptin + metoprolol group was 8.26 ± 2.15 years, while in the sitagliptin group, it was 8.41 ± 2.09 years. The average duration of hypertension in the sitagliptin + metoprolol group was 6.73 ± 1.82 years, while in the sitagliptin group, it was 6.59 ± 1.76 years. No significant differences were observed between groups in terms of age, gender, BMI, duration of diabetes, and duration of hypertension (Table 1, p>0.05).

**Table 1:** Comparison of baseline characteristics.

Group	Sitagliptin + metoprolol (n=26)	Sitagliptin (n=26)	*χ* ^*2*^ */t*	p-Value
Age, year	65.19 ± 7.31	67.08 ± 7.67	−0.907	0.369
Gender (male/female)	20/6	19/7	0.103	0.749
BMI, kg/m^2^	25.83 ± 1.69	25.07 ± 1.54	1.696	0.096
Diabetes duration, years	8.26 ± 2.15	8.41 ± 2.09	0.255	0.800
Duration of hypertension, years	6.73 ± 1.82	6.59 ± 1.76	0.282	0.780

BMI, body mass index.

### Therapeutic effect

Therapeutic efficacy of sitagliptin + metoprolol or sitagliptin was assessed using FPG and HbA_1c_ as blood glucose-related indicators and using SBP and DBP as blood pressure-related indicators. Our data revealed that the levels of FPG (6.14 ± 0.58 vs. 9.36 ± 0.92), HbA_1c_ (6.04 ± 0.13 vs. 8.31 ± 0.24), SBP (124.96 ± 5.30 vs. 152.04 ± 10.31) and DBP (75.31 ± 4.24 vs. 94.27 ± 4.60) were obviously reduced after 12-week treatment in sitagliptin + metoprolol group (Table 2, p*<*0.05). Similarly, the sitagliptin group also showed the decreases of FPG (7.67 ± 0.71 vs. 9.41 ± 0.89), HbA_1c_ (7.18 ± 0.17 vs. 8.22 ± 0.36), SBP (136.85 ± 6.61 vs. 153.27 ± 8.29) and DBP (81.04 ± 4.50 vs. 95.65 ± 5.35) after 12-week treatment (Table 2, p*<*0.05). Of note, the combination therapy yielded significantly greater reductions in FPG (6.14 ± 0.58 vs. 7.67 ± 0.71), HbA_1c_ (6.04 ± 0.13 vs. 7.18 ± 0.17), SBP (124.96 ± 5.30 vs. 136.85 ± 6.61) and DBP (75.31 ± 4.24 vs. 81.04 ± 4.50) compared with sitagliptin alone (Table 2, p*<*0.05), confirming its enhanced therapeutic efficacy in elderly patients with T2DM and hypertension.

**Table 2:** Comparison of the levels of blood glucose and blood pressure between groups before and after treatment.

Indicator		Sitagliptin + metoprolol (n=26)	Sitagliptin (n=26)	*t*	p-Value
FPG, mmol/L	Before treatment	9.36 ± 0.92	9.41 ± 0.89	−0.198	0.844
	After treatment	6.14 ± 0.58^a^	7.67 ± 0.71^a^	−8.5	<0.001
HbA_1c_, %	Before treatment	8.31 ± 0.24	8.22 ± 0.36	1.06	0.295
	After treatment	6.04 ± 0.13^a^	7.18 ± 0.17^a^	−27.043	<0.001
SBP, mmHg	Before treatment	152.04 ± 10.31	153.27 ± 8.29	−0.474	0.637
	After treatment	124.96 ± 5.30^a^	136.85 ± 6.61^a^	−7.153	<0.001
DBP, mmHg	Before treatment	94.27 ± 4.60	95.65 ± 5.35	−1	0.322
	After treatment	75.31 ± 4.24^a^	81.04 ± 4.50^a^	−4.724	<0.001

^a^p<0.05 vs. before treatment in the same group. FPG, fasting plasma glucose; HbA_1c_, glycosylated hemoglobin; SBP, systolic blood pressure; DBP, diastolic blood pressure.

### Immune-inflammation level

The immune-inflammation response related indicators included CRP and NLR. Following 12-week treatment, the sitagliptin + metoprolol group showed significant reductions in the levels of CRP (17.24 ± 1.36 vs. 62.38 ± 6.24) and NLR (5.01 ± 1.14 vs. 8.08 ± 0.54) compared with baseline (Table 3, p*<*0.05). In sitagliptin group, similar but less pronounced reductions were observed for the levels of CRP (35.28 ± 3.36 vs. 61.98 ± 5.47) and NLR (7.34 ± 0.42 vs. 8.07 ± 0.61) (Table 3, p*<*0.05). Notably, the combination therapy was superior to sitagliptin alone in reducing CRP (17.24 ± 1.36 vs. 35.28 ± 3.36) and NLR (5.01 ± 1.14 vs. 7.34 ± 0.42) (Table 3, p*<*0.05).

**Table 3:** Comparison of the levels of CRP and NLR between groups before and after treatment.

Indicator		Sitagliptin + metoprolol (n=26)	Sitagliptin (n=26)	*t*	p-Value
CRP, mg/L	Before treatment	62.38 ± 6.24	61.98 ± 5.47	0.246	0.807
	After treatment	17.24 ± 1.36^a^	35.28 ± 3.36^a^	−25.381	<0.001
NLR	Before treatment	8.08 ± 0.54	8.07 ± 0.61	0.063	0.95
	After treatment	5.01 ± 1.14^a^	7.34 ± 0.42^a^	−9.777	<0.001

^a^p<0.05 vs. before treatment in the same group. CRP, C-reactive protein; NLR, neutrophil to lymphocyte ratio.

### Coagulation function

The coagulation function-related parameters, PT, APTT and Fib, were measured before and after treatment (Table 4). Our data revealed that the values of PT (13.00 (12.00, 13.75) vs. 10.50 (10.00, 11.00)) and APTT (36.73 ± 4.21 vs. 30.81 ± 4.20) were increased, while that of Fib (2.63 ± 0.28 vs. 3.68 ± 0.41) was reduced in the sitagliptin + metoprolol group after 12-week treatment (p*<*0.05). Similar changes were observed in sitagliptin group (PT (12.00 (11.00, 13.00) vs. 10.50 (10.00, 11.75)); APTT (33.92 ± 3.71 vs. 30.42 ± 4.25); Fib (3.04 ± 0.34 vs. 3.52 ± 0.51)) (Table 4, p*<*0.05). The increases of PT (13.00 (12.00, 13.75) vs. 12.00 (11.00, 13.00)) and APTT (36.73 ± 4.21 vs. 33.92 ± 3.71) as well as the decrease of Fib (2.63 ± 0.28 vs. 3.04 ± 0.34) were more remarkable in the sitagliptin + metoprolol group than in the sitagliptin group (Table 4, p*<*0.05).

**Table 4:** Comparison of coagulation function between groups before and after treatment.

Indicator		Sitagliptin + metoprolol (n=26)	Sitagliptin (n=26)	*t/Z*	p-Value
PT, s	Before treatment	10.50 (10.00, 11.00)	10.50 (10.00, 11.75)	−0.235	0.814
	After treatment	13.00 (12.00, 13.75)^a^	12.00 (11.00, 13.00)^a^	−2.148	0.032
APTT, s	Before treatment	30.81 ± 4.20	30.42 ± 4.25	0.328	0.744
	After treatment	36.73 ± 4.21^a^	33.92 ± 3.71^a^	2.552	0.014
Fib, g/L	Before treatment	3.68 ± 0.41	3.52 ± 0.51	1.24	0.221
	After treatment	2.63 ± 0.28^a^	3.04 ± 0.34^a^	−4.745	<0.001

^a^p<0.05 vs. before treatment in the same group. PT, prothrombin time; APTT, activated partial thromboplastin time; Fib, fibrinogen.

### Liver and kidney function

ALT and AST served as liver function markers, whereas Scr and BUN represented renal function indicators. The 12-week treatment resulted in downregulation of ALT (29.28 ± 5.41 vs. 35.85 ± 4.87), AST (30.02 ± 4.01 vs. 34.38 ± 3.57), Scr (116.81 ± 4.86 vs. 125.87 ± 6.47) and BUN (4.94 ± 0.75 vs. 5.98 ± 1.21) in the sitagliptin + metoprolol group (Table 5, p*<*0.05). Also, the sitagliptin group exhibited significant decreases in ALT (32.89 ± 5.19 vs. 35.42 ± 6.53), AST (32.69 ± 4.05 vs. 34.85 ± 4.21), Scr (122.42 ± 4.71 vs. 125.97 ± 5.89) and BUN (5.41 ± 0.73 vs. 5.97 ± 1.10) (Table 5, p*<*0.05). Notably, more remarkable decreases of ALT (29.28 ± 5.41 vs. 32.89 ± 5.19), AST (30.02 ± 4.01 vs. 32.69 ± 4.05), Scr (116.81 ± 4.86 vs. 122.42 ± 4.71) and BUN (4.94 ± 0.75 vs. 5.41 ± 0.73) were detected in the sitagliptin + metoprolol group relative to the sitagliptin group (Table 5, p*<*0.05).

**Table 5:** Comparison of liver and kidney function between groups before and after treatment (mmol/L).

Indicator		Sitagliptin + metoprolol (n=26)	Sitagliptin (n=26)	*t*	p-Value
ALT	Before treatment	35.85 ± 4.87	35.42 ± 6.53	0.27	0.789
	After treatment	29.28 ± 5.41^a^	32.89 ± 5.19^a^	−2.455	0.018
AST	Before treatment	34.38 ± 3.57	34.85 ± 4.21	−0.435	0.666
	After treatment	30.02 ± 4.01^a^	32.69 ± 4.05^a^	−2.384	0.021
Scr	Before treatment	125.87 ± 6.47	125.97 ± 5.89	−0.059	0.954
	After treatment	116.81 ± 4.86^a^	122.42 ± 4.71^a^	−4.227	<0.001
BUN	Before treatment	5.98 ± 1.21	5.97 ± 1.10	0.039	0.969
	After treatment	4.94 ± 0.75^a^	5.41 ± 0.73^a^	−2.266	0.028

^a^p<0.05 vs. before treatment in the same group. ALT, alanine aminotransferase; AST, aspartate aminotransferase; Scr, serum creatinine; BUN, blood urea nitrogen.

### Adverse events

During the 12-week treatment, the adverse events were assessed between groups (Table 6). In sitagliptin + metoprolol group, the adverse events included stomachache (1 case), diarrhea (1 case), nausea (0 case), vomit (0 case), dizzy (0 case), rash (1 case), bradycardia (heart rate at 45–49 times/min, 1 case), cough (1 case), drowsiness (0 case) and tiredness (0 case). In sitagliptin group, the adverse events comprised stomachache (1 case), diarrhea (1 case), nausea (1 case), vomit (2 cases) and dizzy (2 cases). The total incidence of adverse events between groups did not reach statistical significance (Table 6, 19.23 % vs. 26.92 %, p*=*0.510).

**Table 6:** Comparison of adverse events between groups before and after treatment [n (%)].

Adverse event	Sitagliptin + metoprolol (n=26)	Sitagliptin (n=26)	*χ* ^*2*^	p-Value
Stomachache	1 (3.85)	1 (3.85)		
Diarrhea	1 (3.85)	1 (3.85)		
Nausea	0 (0.00)	1 (3.85)		
Vomit	0 (0.00)	2 (7.69)		
Dizzy	0 (0.00)	2 (7.69)		
Rash	1 (3.85)	–		
Bradycardia (heart rate <50 times/min)				
45-49 times/min	1 (3.85)	–		
40-44 times/min	0 (0.00)	–		
<40 times/min	0 (0.00)	–		
Cough	1 (3.85)	–		
Drowsiness	0 (0.00)	–		
Tiredness	0 (0.00)	–		
Total	5 (19.23)	7 (26.92)	0.433	0.510

## Discussion

In this study, the therapeutic effect of sitagliptin + metoprolol on elderly patients with T2DM and hypertension was evaluated. The hypoglycemic effect can be assessed by FPG and HbA_1c_, which presented decreasing trends after treatment, indicating the improvement of T2DM in elderly patients [14], [15], [16]. Of note, sitagliptin can reduce the levels of FPG and HbA_1c_ in T2DM patients [17], 18], consistent with our findings. The hypotensive effect can be evaluated using SBP and DBP, with corresponding decreases in these parameters reflecting improvement of hypertension in elderly patients [19]. Metoprolol can also reduce the levels of SBP and DBP to treat hypertension [20]. Notably, our results indicated that the combination therapy produced significantly greater reductions in FPG, HbA_1c_, SBP, and DBP levels than sitagliptin monotherapy, however, it should be noted that the difference in blood pressure between the two groups in this study may primarily stem from the antihypertensive pharmacological effect of metoprolol itself. Relying solely on blood pressure indicators cannot confirm the synergistic antihypertensive effect of sitagliptin and metoprolol.

Reportedly, the immune-inflammation response may contribute to the pathophysiology of T2DM [21], 22], and a high immune-inflammation level is closely related to the complications in T2DM patients, such as diabetic kidney disease [23]. The common immune-inflammation indicators include CRP and NLR [24]. Existing research has pointed out that sitagliptin exerts anti-inflammatory and glucose-lowering effects on T2DM patients [25], consistent with our observations. Moreover, we found that the decreases of CRP and NLR were more obvious in the sitagliptin + metoprolol group than in the sitagliptin group, indicating superior anti-inflammatory efficacy of the combination therapy in elderly patients with T2DM and hypertension.

The abnormal coagulation function, characterized by the increase of Fib as well as the decreases of APTT and PT, has been documented in T2DM patients [26], 27]. However, the influences of sitagliptin and metoprolol on coagulation function in elderly patients with T2DM and hypertension remain unclear. Our data supported that the coagulation function was improved by sitagliptin intervention, as evidenced by decreased Fib as well as increased APTT and PT, with more pronounced improvements observed upon the addition of metoprolol.

The liver-kidney function in elderly patients with T2DM and hypertension was assessed using ALT/AST and Scr/BUN. The abnormal increases of ALT and AST indicate the underlying injury of liver function [28], while aberrant elevations of Scr and BUN reflect impaired kidney function [29]. Notably, our findings differ from a previous report revealing that sitagliptin can promote the levels of ALT and AST and induce acute liver injury, while mitigating T2DM [30]. In contrast, we found that the levels of ALT and AST were obviously decreased after sitagliptin therapy, together with decreased Scr and BUN levels. A possible explanation for this discrepancy lies in the baseline characteristics of our study population: most patients had mild-to-moderate liver enzyme abnormalities due to long-term glucose and lipid metabolism disorders and hypertension. After effective control of blood glucose and blood pressure, secondary liver function impairment was alleviated. Furthermore, different from most previous studies that used sitagliptin alone, this study applied sitagliptin combined with metoprolol, which provided additional benefits through stable blood pressure control to reduce the pressure on liver blood perfusion, indirectly improving liver enzyme levels. Collectively, these findings suggested that the combined therapy can more effectively improve indicators related to liver and kidney function in elderly patients with T2DM and hypertension.

The safety analysis results of this study showed that one elderly patient in the combination therapy group experienced bradycardia, with a heart rate fluctuating between 45 and 49 beats per minute. Elderly patients had weaker cardiovascular regulatory capabilities and were more sensitive to the heart rate-suppressing effect of beta blockers, making them a high-risk group for bradycardia. The patient with bradycardia did not experience any associated symptoms such as dizziness, blackouts, or chest tightness. The study medication was administered as routine care, without additional protocol-mandated intervention, and the heart rate gradually returned to normal during follow-up. No serious adverse cardiovascular events were documented. The occurrence of this single adverse event was considered attributable to the pharmacological effects of metoprolol and the physiological decline of elderly patients. Importantly, there was no statistical difference in the incidence of adverse events between the two groups, indicating the short-term safety of the combination therapy.

This study has several limitations: ① The control group was only treated with sitagliptin for glucose lowering. The antihypertensive interventions between the two groups were not balanced. The inter-group differences in systolic and diastolic blood pressure only reflected the basic antihypertensive effect of metoprolol, and could not prove the synergistic effect between sitagliptin and metoprolol. Furthermore, the follow-up period was only 12 weeks, lacking long-term follow-up data. The long-term serious cardiovascular and drug-related adverse reactions were not clear; ② In the combination group, there was one elderly patient with bradycardia and a heart rate of 45–49 beats per minute. Although Beta blockers predispose elderly individuals to bradycardia, the small sample size of this study precluded a comprehensive risk assessment; ③ The study did not uniformly and systematically monitor the occurrence of fasting and postprandial hypoglycemia episodes and the dynamic changes in resting heart rate. Metoprolol can mask typical symptoms of hypoglycemia such as palpitations and sweating, and there is a lack of safety data related to hypoglycemia, leading to insufficient safety assessment; ④ Only elderly patients were included, limiting the generalizability of our findings to middle-aged and young adults; ⑤ There were deficiencies in the retrospective medical record collection, as several baseline confounding indicators such as cardiovascular history, and concomitant medications were not recorded, which precluded baseline adjustment; ⑥ No correction for multiple comparison was set, elevating the risk of false positives in the analyses of secondary indicators; ⑦ Given its retrospective non-randomized controlled design, blinded assessment could not be performed, resulting in selection bias. Large-sample, long-term prospective studies should be conducted in the future to avoid the aforementioned deficiencies.

## Conclusions

Short-term observations indicate that, compared to sitagliptin monotherapy for glucose lowering, the combination of sitagliptin and metoprolol can effectively reduce blood glucose and blood pressure levels in elderly patients with type 2 diabetes mellitus (T2DM) complicated by hypertension. The improvement in blood pressure is primarily attributed to the antihypertensive effect of metoprolol. Additionally, this combination therapy can reduce systemic inflammation levels and improve coagulation function as well as liver and kidney function-related indicators. During the 12-week short-term follow-up, there was no statistically significant difference in the overall incidence of adverse reactions between the two groups, indicating that the short-term safety of medication is acceptable. However, due to limitations such as an unbalanced control design, a small sample size, and a short follow-up period in this study, the results only reflect real-world medication observation data from primary communities and cannot confirm the existence of a synergistic therapeutic effect between the two drugs. The long-term efficacy and safety of this combination still require further verification through standardized, balanced grouping, prospective, large-sample studies.
